# The role of insomnia in the vulnerability to depressive and anxiety symptoms in atopic dermatitis adult patients

**DOI:** 10.1007/s00403-023-02538-0

**Published:** 2023-02-07

**Authors:** Federico Salfi, Giulia Amicucci, Michele Ferrara, Daniela Tempesta, Andrea De Berardinis, Andrea Chiricozzi, Ketty Peris, Maria Concetta Fargnoli, Maria Esposito

**Affiliations:** 1 Department of Biotechnological and Applied Clinical Sciences, University of L’Aquila, L’Aquila, Italy; 2 Department of Psychology, Sapienza University of Rome, Rome, Italy; 3 UOSD General and Oncologic Dermatology, San Salvatore Hospital, L’Aquila, Italy; 4 UOC di DermatologiaDipartimento di Scienze Mediche e Chirurgiche, Fondazione Policlinico Universitario A. Gemelli-IRCCS, Rome, Italy; 5 Dermatologia, Dipartimento di Medicina e Chirurgia Traslazionale, Università Cattolica del Sacro Cuore, Rome, Italy

**Keywords:** Atopic dermatitis, Insomnia, Depression, Anxiety, Mediation

## Abstract

Atopic dermatitis (AD) is a common inflammatory chronic skin disease typically associated with atopic comorbidities and other non-atopic conditions such as sleep disturbances, and mood/anxiety disorders. A growing literature proposed a crucial role of sleep disturbances in the development of mental health problems in AD. We tested this assumption by mediation model analyses in adult AD patients.

A total of 57 patients (mean age ± std. dev., 34.28 ± 13.07 years; 27 males; range 18–67 years) diagnosed with AD participated in a cross-sectional study. We evaluated self-perceived severity of AD, insomnia, depression, and anxiety symptoms using validated questionnaires: the Patient-Oriented Eczema Measure (POEM), the Insomnia Severity Index (ISI), the Beck Depression Inventory-second edition (BDI-II), and the Generalized Anxiety Disorder-7 scale (GAD-7), respectively. Two mediation models were performed, testing the mediation effect of insomnia symptoms on the relationship between AD severity and depression (model 1) and anxiety (model 2). AD symptoms, as expressed by POEM, were positively associated with insomnia, depression, and anxiety severity. Insomnia fully mediated the effect of AD severity on depression and anxiety. Specifically, insomnia accounted for 81.64% of the relationship between atopic eczema severity and depression, and for 81.84% of the effect of AD severity on anxiety symptoms. The present study proposed a critical role of insomnia in predisposing adult AD patients to experience depression and anxiety. Early interventions focused on treating sleep disturbances could indirectly be beneficial on mental health of patients with AD, counteracting the onset and exacerbation of anxiety and depression disorders.

## Introduction

Atopic dermatitis (AD) is a common chronic inflammatory skin disease characterized by pruritus, erythema, xerosis, and lichenified lesions [1]. The condition is common among children but can persist into adulthood or have adult onset, affecting 2–10% of the general adult population [1, 2]. Intense night-time itching is typically experienced by AD patients, associated with difficulty falling asleep, frequent nocturnal and early morning awakenings, and consequent daytime sleepiness [2, 3]. Several studies have shown a positive relationship between AD manifestations, including signs and associated itching, and the severity of sleep disturbances [4–7]. A growing literature indicated sleep disturbances as a comorbidity of AD in 33–87% of adult AD patients [2, 3, 8]. Consistently, AD was associated with a higher risk of developing insomnia than adults without AD in a large population-based US study [9].

A wide range of consequences has been attributed to sleep disorders in AD patients [2]. Silverberg et al. showed that insomnia in adults with AD significantly predicts worse overall health status [9]. A study comparing different dermatological disorders demonstrated that sleep disturbances are associated with decreased quality of life [10]. Moreover, AD patients with sleep disturbances are more likely to have difficulties in instrumental activities of daily living (e.g., concentrating, remembering, performing hobbies, doing finances, and driving) [2, 11]. Finally, considering the causal role of insomnia symptoms in the onset and exacerbation of mood and anxiety disorders [12–15], it is not surprising that AD is associated with higher rates of depression [8, 16–19] and anxiety symptoms [8, 18, 19].

However, a recent two-sample Mendelian randomization study involving over 10,000 AD patients reported controversial results [20]. The investigation showed no evidence of a causal role of AD with respect to the development of depressive and anxiety disorders, supporting the existence of an indirect link between AD and psychological measures driven by other concomitant conditions. Consistently, several studies proposed a key role of sleep problems in explaining the well-documented predisposition of AD patients to develop mood and anxiety disorders [2, 8, 18]. Based on these assumptions, it is essential to quantify the contribution of sleep disturbances in the mental health outcomes of patients suffering from AD.

In the present study, we evaluated AD severity, insomnia, depression, and anxiety symptoms in a group of adult patients through validated questionnaires widely used in clinical practice. Then, we investigated the potential determinant role of insomnia symptoms in explaining the tendency of AD patients to experience depression and anxiety symptoms by mediation model analyses. We hypothesized that insomnia could partially explain the relationship between AD severity and measures of depression and anxiety.

## Materials and methods

### Participants and procedure

A descriptive, cross-sectional, case–control, observational study was conducted with consecutive patients referring to the Dermatology Unit, University of L’Aquila (L’Aquila, Italy) with a diagnosis of AD and controls. Patients were recruited in the period between July 2020 and December 2021.

Inclusion criteria were as follows: (i) Adult AD patients, (ii) Patients who voluntarily signed an informed consent on the study objectives, (iii) Patients able to complete questionnaires through a digital medium. Exclusion criteria included: (i) Patients with a previous diagnosis of psychiatric or neurological disorders, (ii) Patients on systemic drugs interfering with sleep, excluding agents for the treatment of AD. Each selected subject participated in the survey using an online platform (*Google forms*).

We collected demographic information (age, sex), and we assessed self-perceived severity of AD and the severity of insomnia, depression, and anxiety symptoms using the following questionnaires: the Patient-Oriented Eczema Measure (POEM) [21], the Insomnia Severity Index (ISI) [22], the Beck Depression Inventory-second edition (BDI-II) [23], and the Generalized Anxiety Disorder scale (GAD-7) [24], respectively. The POEM is a 7-item self-assessed measurement tool for evaluating AD severity (range 0–28). The ISI is a 7-item clinical instrument to assess insomnia symptoms (range, 0–28). The BDI-II is a 21-item inventory used in clinical practice to measure depression symptoms (range 0–63). The GAD-7 is a validated 7-item questionnaire to assess generalized anxiety disorder (range 0–21). For each questionnaire, a higher score denotes more severe conditions. In the present study, all questionnaires approached excellent internal consistency (POEM: Cronbach’s *α* = 0.919; ISI: Cronbach’s *α* = 0.894; BDI-II: Cronbach’s *α* = 0.893; GAD-7: Cronbach’s *α* = 0.888). Harman’s one-factor test did not detect common method bias in our data.

The Internal Review Board of the University of L’Aquila approved the study (protocol n. 35/2020). Online informed consent was obtained from all participants.

### Statistical analysis

Pearson’s correlation analyses were conducted to estimate the bivariate correlations among the main study variables (POEM, ISI, BDI-II, GAD-7).

**Fig. 1:**
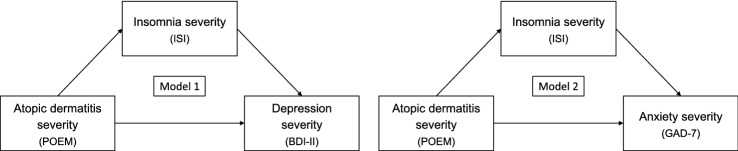
The tested theoretical mediation models. The severity of insomnia symptoms (ISI score) is hypothesized to mediate the effect of atopic dermatitis severity (POEM score) on depressive (BDI-II score) and anxiety symptoms (GAD-7 score). Each model was adjusted for age and sex. *POEM* Patient-Oriented Eczema Measure, *ISI* Insomnia Severity Index, *BDI-II* Beck Depression Inventory-second edition, *GAD-7* Generalized Anxiety Disorder-7

According to the research hypotheses, we run two models to evaluate the mediation effect of insomnia symptoms on the relationship between patient-reported AD severity and psychological dimensions (depression and anxiety) using model 4 of PROCESS macro (version 3.5) for SPSS (version 22.0) [25]. The direction of the effects between AD severity, sleep, and mental health outcome was established based on a consistent meta-analytic literature on longitudinal epidemiological studies supporting a causal role of insomnia in the development of depressive and anxiety symptoms [12, 13], and on a large-scale genome-wide association study that showed no direct relationship between AD and mental health outcomes [20]. Both models were controlled for age and sex. Sex was dummy coded as 0 (female) and 1 (male). A summary of the theoretical models performed is provided in Fig. 1. The mediation effect was ascertained following MacKinnon’s four-step procedure [26]. Firstly, the association between POEM and ISI scores would be significant. Secondly, the relationship between POEM and BDI-II/GAD-7 scores would be significant. Thirdly, the association between ISI and BDI-II/GAD-7 scores would still be significant when controlling for POEM scores. Lastly, the coefficient for the indirect path between POEM and BDI-II/GAD-7 via ISI scores would be significant. We tested the significance of indirect effects using the bias-corrected percentile bootstrap method. Indirect effects were considered statistically significant when the 95% confidence interval (*bootCI*) computed with 5000 resamples did not include zero. Two-tailed* p* values less than 0.05 for bivariate correlations and direct effects of mediation models were considered significant.

## Results

A total of 57 adult patients diagnosed with AD (27 males; mean age of 34.28 ± 13.07 years with a range of 18–67 years; mean disease duration of 23.46 ± 12.12) participated in the study. Ongoing AD treatments were dupilumab (28/57—49.1%), cyclosporine (5/57—8.7%), upadacitinib (2/57—3.5%), methotrexate (2/57—3.5%), antihistamines (10/57—17.5%) and topical treatments (10/57—17.5%). Associated comorbidities were atopic comorbidities (17/57—29.8%), thyroiditis (4/57—7%), alopecia (3/57—5.2%), hypertension (2/57—3.5%), inflammatory bowel disease (2/57—3.5%), metabolic syndrome, migraine, previous stroke, previous thyroid carcinoma and coeliac disease (1/57—1.7%).

AD severity, as assessed by POEM, as well as ISI, BDI-II, and GAD-7 scores are reported in Table 1.

**Table 1:** Bivariate correlations between AD severity (POEM), insomnia (ISI), depression (BDI-II), and anxiety symptoms (GAD-7)

	Mean ± Std. dev	(1)	(2)	(3)	(4)
1) POEM	12.333 ± 8.675	1			
2) ISI	8.386 ± 5.806	0.553***	1		
3) BDI-II	10.772 ± 8.411	0.444***	0.653***	1	
4) GAD-7	7.053 ± 4.580	0.414***	0.637***	0.690***	1

Means ± standard deviations are also reported

*POEM* Patient-Oriented Eczema Measure, *ISI* Insomnia Severity Index, *BDI-II* Beck Depression Inventory-second edition, *GAD-7* Generalized Anxiety Disorder-7

Significance is indicated with asterisks (***p < 0.001)

### Bivariate correlations

Pearson’s correlations (Table 1) showed significant relationships between all variables examined (POEM, ISI, BDI-II, GAD-7). More severe AD symptoms were associated with more severe symptoms of insomnia, depression, and anxiety. Severity of insomnia was positively correlated with symptoms of depression and anxiety. Measures of depression and anxiety were positively correlated.

### Mediation analyses

The regression on mediator (ISI score) was significant (*R*^2^ = 0.312, *F* = 8.010, *p* < 0.001). The significant positive association between POEM and ISI scores was confirmed (*β* = 0.565, *p* < 0.001) adjusting for age and sex.

The regressions on BDI-II and GAD-7 scores without including the mediator were significant (*R*^2^ = 0.229, *F* = 5.254, *p* = 0.003; *R*^2^ = 0.233, *F* = 5.373, *p* = 0.003, respectively). Consistently, POEM scores positively predicted BDI-II and GAD-7 scores (*β* = 0.414, *p* = 0.001; *β* = 0.402, *p* = 0.002, respectively) controlling for demographic factors (age and sex).

**Fig. 2:**
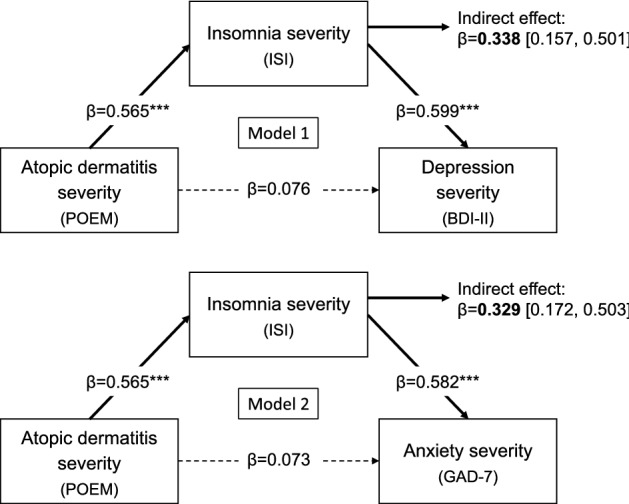
Summary of the results of the two mediation models. The figure reports the standardized coefficients (*β*) of direct and indirect effects, with bootstrapped computed 95% confidence intervals for indirect effects. Asterisks (***p < 0.001) and bold characters indicate significant direct and indirect effects, respectively. *POEM* Patient-Oriented Eczema Measure, *ISI* Insomnia Severity Index, *BDI-II* Beck Depression Inventory-second edition, *GAD-7* Generalized Anxiety Disorder-7

The regressions on BDI-II and GAD-7 scores including the mediator as predictor were significant (*R*^2^ = 0.476, *F* = 11.809, *p* < 0.001; *R*^2^ = 0.466, *F* = 11.356, *p* < 0.001, respectively). Overall results of the mediation models adjusted for age and sex are reported in Fig. 2. Insomnia severity positively predicted depression and anxiety scores (*β* = 0.599, *p* < 0.001; *β* = 0.582, *p* < 0.001, respectively). Meanwhile, the direct relationship between AD severity and psychological measures (BDI-II, GAD-7) disappeared after controlling for the effect of the mediator (*β* = 0.076, *p* = 0.538; *β* = 0.073, *p* = 0.559, respectively). Finally, the indirect effect was significant in both models (*β* = 0.338, 95% bootCI: [0.157, 0.501]; *β* = 0.329, 95% bootCI: [0.172, 0.503], respectively). Taken together, all criteria of MacKinnon’s four-step procedure [26] were satisfied, indicating that ISI scores fully mediated the effect of POEM on BDI-II and GAD-7 scores. Specifically, the severity of insomnia symptoms accounted for 81.64% of the total effect of AD severity on depression, and it explained 81.84% of the relationship between AD severity and anxiety symptoms.

The effects of demographic covariates (age, sex) were not significant in all the above-mentioned regression models (all *p* > 0.10).

## Discussion

AD is a complex disease associated with several atopic and non-atopic comorbidities [8–11]. Among these, sleep disturbances [2, 3, 8] and high odds of insomnia [9] are a substantial component of the disease burden and are often underestimated and undertreated. Our findings are consistent with this literature, as we detected a strong association between AD severity and insomnia symptoms.

AD has been associated with depression, anxiety, and suicidal ideation, but the exact risk estimate is unknown [17, 27]. A recent meta-analysis found that almost 1 in 6 persons with AD had clinical depression, 1 in 4 had depressive symptoms, and 1 in 8 had suicidal ideation [27]. Moderate and severe AD seems particularly associated with markedly worse mental health, and the relationship with AD clinical severity, course, and therapeutical approach is very important in terms of clinician behavior. Our investigation confirmed the relationship between AD severity and depressive [8, 16–19, 27] and anxiety symptoms [8, 18, 19].

Finally, the results of the mediation models supported our research hypothesis, as we suggested that the presence of insomnia symptoms could explain the tendency of AD patients to experience depression and anxiety. Remarkably, our analyses revealed that insomnia explained a large portion of the relationship between AD severity and depression and anxiety symptoms (81.64% and 81.84%, respectively). These results corroborate the idea that sleep disturbances may play a critical role in the development of mood and anxiety disorders in adult AD patients [2, 8, 18].

Our findings are consistent with a recent study [28] showing a mediation effect of sleep disturbances in the association between the POEM score and anxiety and the depression subscales of Hospital Anxiety and Depression Scale (HADS). Albeit this study included a larger clinical sample, sleep problems were evaluated using an unvalidated question asking for the severity of sleep disturbances. In contrast, our results were based on administering a well-established instrument for assessing insomnia symptoms (ISI). In this view, the present study added an important piece to the sleep-mental health puzzle in AD condition.

Effective management of sleep disturbances should rely on accurate identification of the disorder, while there are few therapeutic approaches in clinical practice that specifically target sleep improvement beyond AD treatment [29]. Furthermore, dermatologists should be vigilant and screen for psychological symptoms while treating AD with the most effective agents able to maintain complete disease remission in the long term.

The implementation of specific strategies for treating sleep disturbances could indirectly promote the psychological well-being of patients with AD, counteracting the development of anxiety and depression disorders in the long run. Finally, because of the bi-directional association between sleep disorders and AD [2], we might hypothesize that improvement of insomnia symptoms could have an additional positive effect, alleviating the symptoms of AD itself. Further longitudinal studies are needed to test this hypothesis.

Some limitations of the present study should be acknowledged. AD severity, insomnia, depression, and anxiety symptoms were assessed using self-reported questionnaires. In addition, our findings were obtained in a relatively small clinical sample under different pharmacological treatments, giving a patient-oriented disease evaluation. Finally, we adopted a cross-sectional design, and the direction of the effects can only be hypothesized. Consequently, caution is required considering the bidirectional relationship that could persist between sleep disturbances and depressive/anxiety disorders [30].

In conclusion, our study highlighted the importance of sleep disturbance recognition in clinical practice for screening and prevention of depressive and anxiety symptoms in AD patients, although longitudinal analyses will need to confirm the causal association between the investigated variables.

## Data Availability

The data underlying this article will be shared on reasonable request to the corresponding author.
